# Association between history of abortion and current use of contraceptives among Mongolian Women

**DOI:** 10.1186/s12905-022-01862-3

**Published:** 2022-07-07

**Authors:** Yohane V. A. Phiri, Gunchmaa Nyam, Yuniar Wardani, Doreen Phiri, Kun-Yang Chuang, Hsing Jasmine Chao, Owen Nkoka

**Affiliations:** 1 School of Public Health, College of Public Health, Taipei Medical University, Taipei, Taiwan; 2 Institute for Health Research and Communication (IHRC), P.O Box 1958, Lilongwe, Malawi; 3 Mongolian National University of Medical Sciences, Ulaanbaatar, Mongolia; 4 Faculty of Public Health, Universitas Ahmad Dahlan, Yogyakarta, Indonesia; 5 School of Nursing, College of Nursing, Taipei Medical University, Taipei, Taiwan; 6 Mulanje Mission College of Nursing and Midwifery, P.O Box 45, Mulanje, Malawi; 7 Neuroscience Research Center, Taipei Medical University, Taipei, Taiwan; 8 Institute of Health and Wellbeing, University of Glasgow, Glasgow, UK

**Keywords:** Abortion, Contraceptives, Mongolia

## Abstract

**Background:**

Understanding the factors associated with the adoption of contraceptive methods among women of childbearing age is imperative to improving maternal health outcomes. This study aimed at exploring the association between history of abortion and contraceptive use among Mongolian women.

**Materials and methods:**

We analyzed cross-sectional data of 8373 women aged 15–49 years from the 2018 Mongolian Social Indicator Sample Survey (MSISS). Binary logistic regression models were used to assess the association between abortion history and current contraceptive use while accounting for both individual- and community- level factors.

**Results:**

A total of 4347 (51.92%) and 2525 (30.16%) reported current use of various contraceptive methods and a history of abortion in their lifetime, respectively. Women with a history of abortion were less likely to report current use of contraceptives (adjusted odds ratio (AOR) = 0.72, 95% confidence interval (CI) [0.58–0.89]). Specifically, women with a history of abortion were less likely to report use of IUD (AOR = 0.79, 95% CI [0.71–0.90)]) and injectables (AOR = 0.59, 95% CI [0.41–0.84]). History of abortion was associated with increased likelihood of using abstinence (OR = 1.82, 95% CI [1.31–2.53]) as a contraceptive method.

**Conclusion:**

Our results demonstrated a significant association between history of abortion and contraceptive use. Public health interventions aiming to improve maternal health outcomes through contraceptive use should target women with a history of abortion to improve their uptake.

**Supplementary Information:**

The online version contains supplementary material available at 10.1186/s12905-022-01862-3.

## Introduction

Modern contraceptives are considered the safest method to help couples realize better family planning (FP). Globally, there has been an increase in contraceptive use among women of reproductive age [1]. As an example, between 1994 and 2019, the number of women who employed female sterilization increased by 5.7% from 195 to 219 million. Similarly, the number of women using IUD rose from 133 million in 1994 to 159 million in 2019. Of all the contraceptives, the highest recorded increase was for male condom use (64 million to 189 million) and injection use (17 million to 74 million). As of 2019, a global total of approximately 922 million women aged 15–49 and their partners used contraceptives, with around 44% using modern contraceptive methods.

**Fig. 1:**
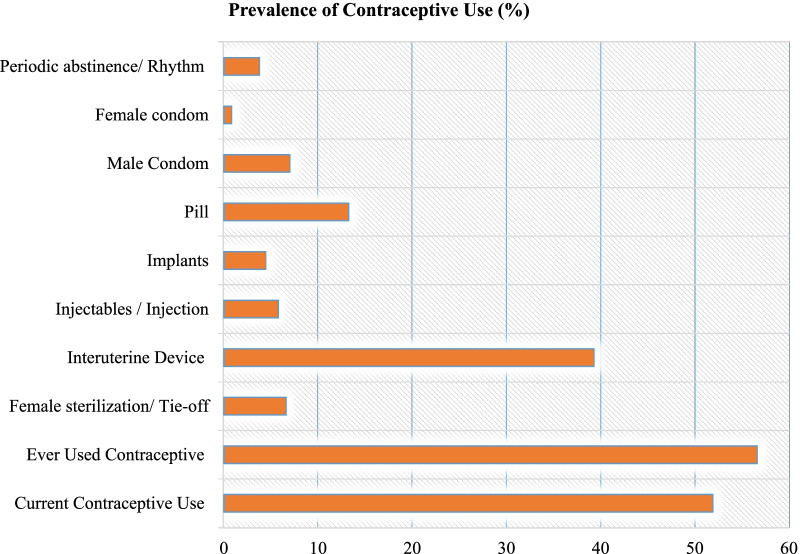
Prevalence of the use of different types of contraceptives among the study participants

For the past five decades, there has been steady progress in contraception research. There is evidence that both hormonal and non-hormonal modern contraceptives contribute to improved women’s health through, among others, preventing unplanned pregnancies and ensuring optimum birth spacing [2]. Contraceptive prevalence rate (CPR) is an important indicator for measuring access to reproductive health services [3]. Unmet need for FP is another important indicator for the gap in terms of women’s reproductive intentions and their contraceptive behaviour [4]. Women are said to have an unmet need for FP if they want to stop or delay/postpone childbearing but are not using any method of contraception [5]. Despite the availability of various contraceptive methods and registered increasing global contraceptive use, communities in both the developed and developing countries continue to register high rates of unintended and unwanted pregnancies which contribute to a higher prevalence of abortions [6–8]. Annually, across the globe, 81 million unintended pregnancies are recorded with up to 19 million women experiencing unintended pregnancies resorting to unsafe abortions in developing countries [6, 8, 9]. Unmet needs for family planning and ‘contraceptive failure’ are reported the main cause of most of the reported unintended pregnancies in women [2]. Even though nearly 50% of reported unintended pregnancies occur in contraceptive users, only 10% of these pregnancies are attributed to true method failure [4, 5, 10, 11]. Therefore, the important role that contraceptives play in preventing unintended pregnancies cannot be downplayed. Despite the increase in the use of contraceptives among Mongolian women recorded between 1990 and 2000, there has, recently, been a drastic drop. The CPR in Mongolia declined from 69% to 40.7% between 2003 and 2019 while unmet need for modern contraceptive methods among women of childbearing age is pegged at 21.2% [3, 12].

Several factors contribute to the recent decreases in contraceptive use in developing countries. Access to family planning methods and several individual and community level socioeconomic factors remain prominent determinants of contraceptive use in developing countries [13, 14]. Understanding the various factors determining the adoption of any contraceptive uptake among women of childbearing age is important to improving contraceptive uptake. One important factor that has been associated with the use of contraceptives in both developed and developing countries is history of abortion [15–17].

In Mongolia, it is estimated that 24% of the women in the reproductive age group use abortion as a birth prevention method [18–20]. Mongolia has a pro-child and population growth policy, but no limitations and access to contraceptives or abortions are set [19]. Both married and unmarried women, adolescents included, are allowed access to contraceptives for free as a FP choice. Furthermore, abortion services are provided to women of all age groups to terminate unwanted pregnancies. Abortion has been legal in Mongolia since 1989, with legal boundaries established [21]. Access to abortion services is currently provided by secondary, tertiary level hospitals and private clinics upon request by those in need [19]. In Mongolia, there does not appear to be access limitations to contraception or abortions and yet contraceptive uptake continues to decline while the use of abortion as a birth prevention method increases. Therefore, it is evident that dissemination of information on available contraceptives methods and their use seems to be lacking. Limited provision of information regarding contraceptives use is a burden to help women prevent unwanted pregnancies, which is a higher risk for abortion in developing countries [22, 23]. To improve the use of contraceptives and reduce abortion, there needs to have public health programs that promote awareness and the use of contraceptive methods.

Access to long-acting reversible contraceptives (LARCs) remain at secondary and tertiary clinics only; with no policies detailing male and female sterilization (tubal ligation) [18–20]. Additionally, contraceptive use in Mongolia varies across several socio-economic factors such as education, wealth index, and area of residence [20]. According to a report by MONFEMNET National Network [3] on the quality of reproductive health in Mongolia, women in urban areas were reported to have higher unmet needs for contraceptives as compared to rural-based women [19–21]. Therefore, establishing the association between having a history of abortion and the use of contraceptives is paramount while accounting for socioeconomic factors in Mongolia. Results from such a study would be essential for policymakers to design and implement effective interventions or programs to increase the uptake of contraceptive methods among Mongolian women of reproductive age. Increase in uptake of contraceptives and a decrease of abortion use will effectively minimize health risks attributed to elective abortions.

It is imperative to acknowledge that contraceptive methods have been associated with various health risks. Modern contraceptives such as pills have been associated to health risks such as cardiovascular and metabolic diseases [24]. Despite LARCs reported to be the safest, health risks such as ectopic pregnancies amongst those who employed implants have been reported [25]. The use of IUD is reported to increase the risk of spontaneous abortion, tubal infertility, uterine perforation and pelvic inflammatory diseases [24, 26]. Nevertheless, unintended pregnancy is associated with increased risk for complications for both the mother and baby [4, 24]. The risks of using modern contraceptives have been reported in less than 2% of the users compared to the risks of not using any contraception or abortion in early pregnancy [24]. Using no contraception and early pregnancy abortion carries a higher risk of death as compared to usage of any of the contraceptive methods. Literature shows that method failure rate associated with modern contraceptive use are consistently low despite the variability of user failure rates [27]. Apart from prevention of unintended pregnancy, contraceptive methods have a range of other benefits. A reduction in pregnancy related to morbidity, mortality and complications from seeking abortion in early pregnancy have been reported [24].

Despite the various projects and policy implementation undertaken by the Mongolian government regarding FP service provision, an understanding of factors associated with contraceptive use is needed owing to the observed decline in CPR, a relatively high unmet need for modern contraceptives, and increasing number of abortions in recent years. According to our literature review, there are no studies that have examined the association between the history of abortion and contraceptive use in Mongolia. Therefore, using national representative data, this study aimed to examine the association between history of abortion and contraceptive use among Mongolian women of reproductive age.

## Methodology

### Study design, setting, sampling and data collection

This was a cross-sectional survey that analyzed secondary data from the 2018 Mongolian Social Indicator Sample Survey (MSISS) [28]. Mongolia has a population of about 2.8 million, with close to 69% of its population believed to occupy the capital, Ulaanbaatar [3].. The MSISS complements the earlier Multiple Cluster Indicator Surveys (MCIS) conducted every five years dating back to 1996. The MSISS was first introduced in 2013 with support from United Nations Children’s Emergency Fund (UNICEF) and United Nations Population Fund (UNFPA). A total of 14,500 households were sampled. All women aged 15–49 years from the sampled provinces were eligible to participate in the survey. A total of 11,737 women were interviewed. In the current study, participants with complete information on all the selected variables were analyzed (n = 8373).

Information on the design, methodology, and sampling techniques of the MSISS have been detailed elsewhere [28]. In brief, the MSISS is a household survey with the final sampling units being individuals at each enlisted household. The 2018 MSISS was designed to cover the largest number of indicators than other previous surveys. The 2018 survey covered five geographical regions (Eastern, Western, Central, Khangai and Ulaanbaatar) both in rural and urban areas aimed at providing a large number of estimates of indicators on the situation of women, children and men. The selection of the survey sample was based on a two-stage stratified cluster sampling technique, employing the 2017 Population and Household Database sampling frame. A total of 8 targeted provinces/districts were singled out from the five regions (Bayan-Ulgii, Bay ankhongor, Gobi-Altai, Zavkhan, Umnugovi, Khuvsgul, Bayanzurkh and Nalaikh) from which samples were drawn.

Data was collected through the completion of questionnaires using computer assisted personal interview. Paper and pencil interviewing was employed during pretesting, which resulted in the modification of wording and coherence of a couple of items in the questionnaire. All the people involved in the data collection went through rigorous training on interviewing techniques, contents of the questionnaire and other vital elements. The MSISS questionnaire was designed to collect data on characteristics of households, women, men and children. The data used in this study comprised of self-reported responses. The questionnaire had several sections including women’s socio-demographic information, contraception use, unmet need for contraception, access to mass and social media and or technology, fertility, miscarriage, stillbirth and abortion, maternal and newborn health, attitudes towards domestic violence, adult function and many more. The data extracted for this study was obtained from the women’s socio-demographic information, contraception use and miscarriage, stillbirth and abortion sections.

### Study variables

#### Outcome measure

The outcome variable was current use of contraceptives by women of reproductive age (15–49 years). Contraception methods were defined as devices, medications or methods used to avoid pregnancy [29]. First, we assessed overall contraceptive use (i.e., whether participant reported to be using any contraceptive method (yes/no)). Women were asked the following question “*Are you currently doing something or using any method to delay or avoid getting pregnant?*”. Second, we assessed the use of specific contraceptive method. Participants were asked to report the type of contraceptive method using the following question “w*hat type of method are you using?*”. This was a “yes/no” question. Participants reported using different types of contraceptive methods (i.e. permanent non-reversible methods [male and female sterilization], long acting reversible contraception (LARC) [IUD or Implants], any other modern contraceptive methods [i.e., injections, pills, male or female condoms, foam/jelly], and traditional or natural methods [lactational amenorrhea method (LAM), periodic abstinence/rhythm/calendar, withdrawal] or any other method) they were using at the time of the interview. We created a variable ‘use of specific contraceptive method’ with nine mutually exclusive categories (i.e., ‘0’ no contraceptive use, ‘1’ female sterilization, ‘2’ IUD, ‘3’ injection, ‘4’ implants, ‘5’ pills, ‘6’ male condom, ‘7’ female condom, ‘8’ abstinence). Even though the question regarding contraceptive use may have been affected with the potential of social desirability bias (in which women may have wanted to report use of contraceptives when they are not using hence resulting in overestimation of contraceptive use), the data collectors were well trained to assure participants of the confidentiality of their responses to ensure participants provide accurate information.

#### Primary independent variable

Our main independent variable was history of abortion (Yes or No). During the survey, women of reproductive age were asked whether they had ever experienced any case of their pregnancy ending up with miscarriage, stillbirth, missed abortion or abortion [28]. The responses were self-reported based on the respondent’s total lifetime number of history of abortions. The variable was coded ‘Yes’ (for those with a history of abortion) and ‘No’ (for those with no abortion history).

#### Covariates

Variables considered as covariates were selected and classified as individual or community- level factors based on literature [30, 31]. Based on our outcome of interest, history of abortion, missing cases from each of the covariates used in this study were dropped. Age of the women (15–19, 20–24, 25–34, 35+), their marital status (married, formerly married/divorced, never married), highest educational level (secondary[lower/upper], vocational or training center, and university/institute/collected), age at first marriage (10–19, 20–29, 30+), currently pregnant (yes/no), ever given birth (yes/no), alcohol use (yes/no), age at first use of alcohol (10–19, 20–29, 30+, Never), the total number of children (Less or equal 2, Less or equal 4, Equal or more than 5, None) and age of the husband (15–24, 25–34, 35+) were the sociodemographic and individual-level factors included in this study. Community-level factors included were area of residency (rural/urban), area of origin (Khangai, Central, Eastern, Ulaanbaatar, Western), ethnicity (Khalkh, Kazakh, Other), religion (Buddhist, Islam, Other, No Religion), and wealth index score (Richest, Fourth, Middle, Second, Poorest).

### Statistical analysis

Chi-square test was used to examine the distribution of study characteristics according to history of abortion and contraceptive use, respectively. We used binary logistic regression to report the association between the outcome and the independent variables. Variables assessed in the current analysis were selected based on their importance in literature [30, 31]. Univariable models were constructed and variables with a *p* < 0.1 were included in the multivariable models [32]. In our final analyses, four models were run. Model 1 was the unadjusted model between history of abortion and contraceptive use. In models 2 and 3, we adjusted for individual and community level factors, respectively. To check for multicollinearity of the models, variance inflation factor and tolerance were used with VIF < 10 and tolerance > 0.1 indicating no multicollinearity problems in our models. Furthermore, we used receiver-operating characteristic (ROC) analysis to compare and evaluate the accuracy of the four statistical models employed [33, 34]. The higher the value of the AUC or the larger the area under curve, the better the performance of the model. The strength of association was reported as adjusted odds ratio (AOR) and their 95% confidence intervals. The statistical significance was set at *p* < 0.05. All analyses were carried out using SAS 9.4 (SAS Institute Inc., Cary, NC, USA).

### Ethical consideration

The MSISS was approved by order number A/67 2018 of Chairperson of NSO in 2018.The order A/67 2018 had details relating to the potential risks and mitigation of same through the lifecycle of the survey under its Protection Protocol. Informed consent was obtained before commencement of the survey from each of the participants or their legal guardian. The participants were assured of the confidentiality and anonymity of any information they had provided. The survey was conducted in accordance with approved guidelines and regulations.

## Results

### CPR

A total of 8373 women were analyzed. Figure 1 displays the prevalence of the use of contraceptive methods per type among Mongolian women. Our results indicated that of the surveyed women, 51.92% reported using at least one contraceptive method. Furthermore, IUD was the most used contraceptive method (39.29%) followed by pills (13.25). The least used contraceptive method was female condoms (n = 33). Most of the participants in the survey indicated to use LARCs (64.25%) as compared to permanent non-reversible method (6.56%). Among those that used any of the eight contraceptive methods consider for analysis in this study, 49.50% reported having a history of abortion.

### Study characteristics by history of abortion and contraceptive use

The results in Table 1 revealed that 30.16% (n = 2525) of the surveyed women reported having experienced an abortion. Approximately 51.56% of those who did not use contraceptives reported of an abortion history. There were significant differences (*p* < 0.05) between the considered covariates and history of abortion except for age at first marriage (*p* = 0.60). Women who were currently married (88.07%), had ever given birth (95.33%), started using alcohol at the age of 30+ (61.17%), resided in urban areas (60.4%) and were from the Khalkh ethnic group (80.28%) had a higher prevalence of history of abortion.

**Table 1:** Distribution of study characteristics by history of abortion

Variable	Abortion				*P* value^c^
	Yes		No		
	n = 2525	%	n = 5848	%	
Age (years)					** < 0.001**
15–19	13	0.51	57	0.97	
20–24	86	3.41	503	8.6	
25–34	892	35.33	2171	37.12	
35+	1534	60.75	3117	53.3	
Marital status					** < 0.001**
Currently married	2223	88.07	5023	85.89	
Formerly married/divorced	233	9.23	537	9.18	
Never married	68	2.69	288	4.92	
Highest educational level					** < 0.001**
Lower/upper secondary school	984	39.52	2847	49.8	
Vocational/training center	327	13.13	858	15.01	
University/institute/college	1179	47.35	2012	35.19	
Age at first marriage (years)					
0.6031					
10–19	751	30.58	1651	29.69	
20–29	1597	65.02	3644	65.54	
30+	108	4.4	265	4.77	
Currently pregnant					**-**
Yes	0	0	0	0	
No	2513	100	5820	100	
Ever given birth					** < 0.001**
Yes	2407	95.33	5829	99.68	
No	118	4.67	19	0.32	
Alcohol use					** < 0.001**
Yes	2171	86.01	4221	72.23	
No	353	13.99	1623	27.77	
Age at first use of alcohol					** < 0.001**
10–19	19	0.88	52	1.23	
20–29	566	26.07	889	21.06	
30+	1328	61.17	2683	63.56	
Never	258	11.88	597	14.14	
Total number of children					** < 0.001**
Less or equal 2	116	4.59	17	0.29	
Less or equal 4	1199	47.49	3037	51.93	
Equal or more than 5	1060	41.98	2304	39.4	
None	150	5.94	490	8.38	
Age of husband (years)					** < 0.001**
15–24	33	1.48	260	5.18	
25–34	725	32.61	1776	35.36	
35+	1465	65.9	2987	59.47	
Area of residence					** < 0.001**
Urban	1525	60.4	2731	46.7	
Rural	1000	39.6	3117	53.3	
Region of origin					** < 0.001**
Khangai	510	20.2	1635	27.96	
Central	476	18.85	1298	22.2	
Eastern	457	18.1	881	15.06	
Ulaanbaatar	320	12.67	864	14.77	
Western	762	30.18	1170	20.01	
Ethnicity					** < 0.001**
Khalkh	2023	80.28	4455	76.55	
Kazakh	178	7.06	583	10.02	
Other	319	12.66	782	13.44	
Religion					** < 0.001**
Buddhist	1001	39.69	2378	40.85	
Islam	1235	48.97	2728	46.86	
Other	160	6.34	519	8.91	
No religion	126	5	197	3.38	
Wealth Index Score					** < 0.001**
Richest	517	20.48	1981	33.87	
Fourth	501	19.84	1443	24.68	
Middle	534	21.15	1057	18.07	
Second	569	22.53	812	13.89	
Poorest	404	16	555	9.49	
Current contraceptive use					** < 0.001**
Yes	1223	48.44	3124	53.42	
No	1302	51.56	2724	46.58	

^c^*p* value from Chi-Square tests, bold means significant i.e., *p* < 0.05

About 51.92% of the reported using of contraceptives at the time of the survey (Table 2). Among others, a high proportion of women aged 35+ (52.7%), whose husbands were aged 35+ (60.68%), and currently married (92.04%) reported using contraceptives. Furthermore, a high proportion of women with ≤ 4 children (47.78%), rural residents (54.5%) and had married aged 20–29 years (66.92%) indicated using contraceptives. Similarly, a high proportion of contraceptive users was observed among women from the Khalkh ethic group (77.19%), who had ever given birth (99.42%) and consumed alcohol (76.2%).

**Table 2:** Distribution of study characteristics by contraceptive use

Variables	Current contraceptive use				*P* value^c^
	Yes		No		
	n = 4347	%	n = 4026	%	
Age (years)					** < 0.001**
15–19	20	0.46	50	1.24	
20–24	275	6.33	314	7.8	
25–34	1761	40.51	1302	32.34	
35+	2291	52.7	2360	58.62	
Marital status					** < 0.001**
Currently married	4001	92.04	3245	80.62	
Formerly married/divorced	234	5.38	536	13.32	
Never married	112	2.58	244	6.06	
Highest educational level					** < 0.001**
Lower/upper secondary school	2100	49.35	1731	43.8	
Vocational/training center	587	13.8	598	15.13	
University/institute/college	1568	36.85	1623	41.07	
Age at first marriage (years)					** < 0.001**
10–19	1240	29.28	1162	30.73	
20–29	2834	66.92	2407	63.66	
30+	161	3.8	212	5.61	
Currently pregnant					
Yes	0	0	0	0	-
No	4333	100	4000	100	
Ever given birth					** < 0.001**
Yes	4322	99.42	3914	97.22	
No	25	0.58	112	2.78	
Alcohol use					**0.6720**
Yes	3310	76.2	3082	76.59	
No	1034	23.8	942	23.41	
Age at first use of alcohol					**0.0655**
10–19	37	1.12	34	1.1	
20–29	754	22.78	701	22.74	
30+	2112	63.81	1899	61.62	
Never	407	12.3	448	14.54	
Total number of children					< **0.001**
Less or equal 2	24	0.55	109	2.71	
Less or equal 4	2077	47.78	2159	53.63	
Equal or more than 5	1904	43.8	1460	36.26	
None	342	7.87	298	7.4	
Age of husband (years)					**0.0003**
15–24	135	3.37	158	4.87	
25–34	1438	35.94	1063	32.76	
35+	2428	60.68	2024	62.37	
Area of residence					** < 0.001**
Urban	1978	45.5	2278	56.58	
Rural	2369	54.5	1748	43.42	
Region of origin					** < 0.001**
Khangai	1128	25.95	1017	25.26	
Central	1020	23.46	754	18.73	
Eastern	663	15.25	675	16.77	
Ulaanbaatar	653	15.02	531	13.19	
Western	883	20.31	1049	26.06	
Ethnicity					**0.1291**
Khalkh	3337	77.19	3141	78.19	
Kazakh	421	9.74	340	8.46	
Other	565	13.07	536	13.34	
Religion					
Buddhist	1756	40.51	1623	40.48	**0.1392**
Islam	2060	47.52	1903	47.47	
Other	369	8.51	310	7.73	
No religion	150	3.46	173	4.32	
Wealth Index Score					
Richest	1472	33.86	1026	1026	** < 0.001**
Fourth	1011	23.26	933	933	
Middle	752	17.3	839	839	
Second	657	15.11	724	724	
Poorest	455	10.47	504	504	

^c^*p* value from Chi-Square tests, bold means significant i.e., *p* < 0.05

### Association between history of abortion and contraceptive use

In our regression analyses, model 4 was a better fit model as compared with the other models based on the ROC test and therefore, results for model 4 are reported here. Additionally, no multicollinearity problems were observed with the models as all had VIF values < 10 and tolerance > 0.1 (Additional file 1: Table S2). Having a history of abortion was associated with reduced odds of reporting current use of contraceptives (AOR = 0.72, 95% CI [0.58–0.89]). Furthermore, history of abortion remained significantly associated with reduced odds of using injection (AOR = 0.59, 95% CI [0.41–0.84]) and IUD (AOR = 0.79, 95% CI [0.71–0.90]) when both individual and community level factors were adjusted. Women that reported having a history of abortion were more likely to use abstinence as a contraceptive method compared with those that did not report any abortion history (AOR = 1.82, 95% CI [1.31–2.53]) (Table 3). Additional file 1: Table S1 lists the association between a wide range of individual- and community-level factors and contraceptive use.

**Table 3:** Association between contraceptive use and history of abortion

	Current contraceptive use	Female sterilization	IUD	Injection	Implants	Pills	Male condoms	Female condoms	Abstinence
	OR (95% CI), *P* value	OR (95% CI), *P* value	OR (95% CI), *P* value	OR (95% CI), *P* value	OR (95% CI), *P* value	OR (95% CI), *P* value	OR (95% CI), *P* value	OR (95% CI), *P* value	OR (95% CI), *P* value
Model 1	0.82 (0.75–0.90), 0.000**	0.94 (0.72–1.21) 0.610	0.70 (0.63–0.78) 0.000**	0.47 (0.34–0.65) 0.000**	1.04 (0.76–1.42) 0.813	1.05 (0.77–1.12) 0.4264	1.35 (1.07–1.72) 0.0135**	1.20 (0.59–2.44) 0.623	2.26 (1.64–3.11) 0.000**
Model 2	0.87 (0.79–0.96) 0.007**	0.916 (0.70–1.20) 0.5282	0.75 (0.67–0.84) 0.000**	0.51 (0.36–0.73) 0.000**	1.27(0.91–1.75) 0.156	0.97 (0.80–1.17) 0.751	1.30 (1.01–1.68) 0.040*	1.24 (0.60–2.56) 0.566	1.96 (1.41–2.72) 0.000**
Model 3	0.89 (0.81–0.98) 0.015*	1.10 (0.84–1.44) 0.491	0.76 (0.68–0.85) 0.000**	0.60 (0.43–0.84) 0.003*	1.05 (0.76–1.44) 0.767	0.97 (0.80–1.17) 0.754	1.24 (0.97–1.59) 0.087	1.13 (0.55–2.34) 0.735	1.94 (1.40–2.69) 0.000**
Model 4	0.72 (0.58–0.89) 0.003**	0.95 (0.72–1.26) 0.730	0.79 (0.71–0.90) 0.000**	0.59 (0.41–0.84) 0.004**	1.22 (0.87–1.69) 0.247	0.98 (0.81–1.19) 0.836	1.27 (0.98–1.64) 0.070	1.17 (0.56–2.45) 0.678	1.82 (1.31 2.53) 0.000**

Model 1: Unadjusted model of the association between history of abortion and contraceptive use

Model 2: Model 1 adjusted for individual-level characteristics (i.e., Age, Marital Status, Highest Educational Level, Ever Given Birth)

Model 3: Model 1 adjusted for community-level factors (i.e., Area of Residence, Region of Origin, Ethnicity, Religion, Wealth Index Score)

Model 4: Model 1 adjusted for both individual and community level characteristics

*p values less than 0.05

**p value less than 0.01

## Discussion

This is the first study to use the MSISS national representative data and examine the association between history of abortion and contraceptive use in Mongolia. In this study, we observed a strong link between having a history of abortion and contraceptive use among Mongolian women. Our study findings reveal that women with history of abortion were more likely to report use of abstinence as a contraceptive method. Specifically, we observed reduced reporting current use of IUD and injection among women who reported history of abortion.

Our current study demonstrates that in Mongolia 51.92% of women used any available modern contraceptive method and only 3.78% used abstinence. The most commonly used modern contraceptive was IUD (39.29%) and the least being female condoms (0.81%). Compared to global data, our study demonstrated a higher IUD prevalence rate by 20.29% amongst women aged 15–49 years in Mongolia as estimated by the United Nations, Department of Economic and Social Affairs, Population Division in 2019 [1]. A 2015 Mongolian report by MONFEMNET National Network and Family Planning 2030 (FP2030) indicated that the CPR was at 49% for all women and 40% for those around reproductive age in Mongolia as of 2015 [3, 35]. Our findings suggest that CPR in Mongolia is below the average 60% rate reported in other East Asian countries [1]. Mongolia’s CPR has been on the decline despite the continued rise in CPR globally [1, 3].Hence, studies to understand factors associated with contraceptive use are warranted and this current study partly addresses that.

The overall abortion prevalence rate (APR) in this study was 30.16% (2525) of which 1223 (48.44%) reported using contraceptives. Similar results were reported in a study conducted in Angola where 82.76% of women who had access to contraceptives reported a history of abortion [31]. Despite the higher APR among those who reported use of contraceptive in our study, the proportion was slightly higher among those who didn’t use contraceptives (51.56%). The relationship between APR and CPR remains unclear. A meta-analysis conducted in 14 countries in Africa, Asia and Europe, including studies with national representative data, showed a bidirectional relationship between APR and CPR [30]. A simultaneous rise in abortion and contraceptive usage were observed in six countries (Cuba, Denmark, Netherlands, the United States, Singapore and the Republic of Korea) while an inverse relationship was observed among the other seven countries (Kazakhstan, Kyrgyz Republic, Uzbekistan, Bulgaria, Turkey, Tunisia and Switzerland). The paradox is also highlighted in a study that examined data between 1978 and 2010 in France and a report from Georgia compiling findings from Reproductive Health Surveys between 1999 to 2005 [36, 37]. It is, therefore, critical to explicate the association between history of abortion and contraceptive use among Mongolian women.

### The link between history of abortion and contraceptive use

Women who reported having a history of abortion had a higher likelihood of using abstinence as a contraceptive method. On the other hand, women who reported a history of abortion were less likely to report use of IUD, injection, and contraceptive use in general. Several factors may explain the relationship observed in these findings. On the association between history of abortion and likelihood of using abstinence as a contraceptive, these results are consistent with findings in other previous studies which reported potential association between abortion and contraceptives use among sexually active women. Trussell and Wynn [38] demonstrated that all contraceptive methods, under normal adherence circumstances, are likely to have low or modest failure rates. Such methods include condom use, abstinence, withdrawal and many other. Methods such as condoms, periodic abstinence and withdrawal require skills, memory, and discipline; hence failure rates recorded from such methods are often much higher [39]. As our study is a cross-sectional survey, we cannot determine whether it is the use of abstinence that led to failure and then unwanted pregnancies resulting in abortion. However, evidence compiled by Bradley et al. [11] from 15 Demographic Health Surveys (DHS’s) indicated abstinence, condoms, and withdrawal were associated with the risk of unintended pregnancy by month number twelve of use. It was further established that abstinence, withdrawal and condoms use attributed 19%, 17% and 9% respectively of unintended pregnancies in the data gathered from the fifteen countries. Nevertheless, future longitudinal study designs are needed to determine the direction of the relationship between having a history of abortion and contraceptive use.

Our results further revealed the vital role played by both individual and community level factors when examining the association between history of abortion and contraceptive use. Evidence of the role of both individual and community level factors on contraceptive use is well documented in developing countries [40, 41]. In this study we observed that age of the women, their marital status, level of education, and whether they had given birth before were the individual factors associated with contraceptive use. Furthermore, the women’s areas of residence, region of origin and their wealth index score were the community level factors associated with contraceptive use. In Mongolia, evidence of several sociodemographic and community level factors affecting the uptake and distribution of contraceptives have been established [42]. Our results align to these previous findings and reveal that individual and community level factors are significant determinants of contraceptive uptake. Previous evidence shows that urban residents are more likely to have a higher contraceptive uptake rate as compared to rural residents [43–46]. On the contrary, in this study, women residing in urban areas were less likely to report using contraceptives compared to women residing in rural areas. This was further evidenced by higher CPR among women from rural areas as compared to those residing in urban areas. It is indicated that there is no explanation as to why rural Mongolian women have a higher CPR [3, 20]. Perhaps, this could be attributed to the focus of local contraceptive programs in rural than urban areas [47]. Gereltuya et al. [42] affirms that community level factors are responsible for contraceptive access variations among *bag, soms (the second administrative unit in rural area) and horoos* in Mongolia. These variations have partially resulted in decreased use of contraceptives and increased unmet need for contraception, resulting in higher rates of unintended abortions [3].

### Strengths and limitations

Our study had both of strengths and limitations. The data used in this study comes from a national representative survey hence the results can be generalized to Mongolian women of childbearing age. The data used in this study was from a cross-sectional survey, therefore, we could not establish the causal association between the variables considered. The study relied on self-reported abortion which may have been underreported (due to perceived social stigma relating to abortion) and self-reported contraceptive use (which may have been overreported) resulting in misclassification bias. However, access to abortion services and contraceptive use has been legal to women of all age groups in Mongolia since 1989 hence, stigma may be unlikely, and participants may have provided information without worrying about legal restrictions or implications. In case of abortion underreporting, our results would not decrease if there were more abortions compared with the ones reported. Additionally, the interviewers were experienced and trained to collect such information by building confidence, trust, and good rapport with the participants to respond to such questions. Nevertheless, results should be carefully interpreted owing to these potential sources of bias.

## Conclusion

Our results demonstrated a significant association between history of abortion and contraceptive use. Future studies should prospectively examine contraceptive use and abortion history to determine the temporal trend of this association. Additionally, rural–urban differences should be taken into consideration when designing family planning programs aimed at improving the use of modern contraceptives methods.

## Supplementary Information

**Additional file 1. Table S1**. Association between current contraceptive use and individual and community-levelcovariates.** Table S2**. Results for Model Multicollinearity Test.

## Data Availability

The data used in this study was accessed from, with permission, from Mongolia National Statistical Office. The data is publicly accessible upon request and registration through the website http://web.nso.mn/nada/index.php/catalog/SISS/dataset.

## Abbreviations

AOR: Adjusted odds ratios
CI: Confidence interval
MSISS: Mongolian Social Indicator Sample Survey
CPR: Contraceptive prevalence rate
APR: Abortion prevalence rate
FP: Family planning
LARCs: Access to long: acting reversible contraceptives
MCIS: Multiple Cluster Indicator Surveys
DHS: Demographic Health Surveys
