# Supplementary material for: Association between history of abortion and current use of contraceptives among Mongolian Women
Source: BMC Womens Health. 2022 Jul 7;22:279. doi: 10.1186/s12905-022-01862-3 (PMC9261006; doi:10.1186/s12905-022-01862-3)
Supplement: Supplementary file 1 — Additional file 1. Table S1. Association between current contraceptive use and individual and community-levelcovariates. Table S2. Results for Model Multicollinearity Test. [file 12905_2022_1862_MOESM1_ESM.docx]

| SUPPLEMENTARY TABLE  Table S1: Association between current contraceptive use and individual and community-level covariates | | | | | |
| --- | --- | --- | --- | --- | --- |
| Variables | **N** | **OR (95% CI)** | **P-Value** | **AOR (95% CI)** | **P-Value** |
| Age  15-19  20-24  25-34  35+ | 11737 | 0.04 (0.03-0.06)  0.50(0.43-0.58)  1.28 (1.17-1.40)  Ref | <0.001  0.002  <0.001 | 1.09 (0.40 2.98)  1.21(0.87-1.67)  1.56 (1.31 1.86)  Ref | 0.806  0.962  **0.05** |
| Marital Status  Currently married  Formerly married/Divorced  Never married | 11687 | 11.79 (10.07-13.82)  4.210 (3.394-5.222)  Ref | <0.001  0.021 | 2.3 (1.86-2.92)  1.04 (1.01-1.07)  Ref | **<.0000**  **0.01** |
| Highest Educational Level  Lower/Upper Secondary School  Vocational/Training Center  University/Institute/College | 10593 | 1.039 (0.954-1.13)  1.073 (0.946-1.22)  Ref | 0.939  0.389 | 1.29 (1.14-1.46)  1.11 (0.93 1.32)  Ref | **0.002**  0.810 |
| Age at First Marriage  10-19  20-29  30+ | 9443 | 1.45 (1.17-1.80)  1.57 (1.27-1.93)  Ref | 0.029  <0.001 | 0.98 (0.74-1.28)  1.16 (1.01 -1.50)  Ref | 0.248  **0.035** |
| Ever Given Birth (Ref=No) | 10794 | 17.95 (14.75-21.85) | <0.001 | 6.34 (4.83-8.32) | **<0.000** |
| Alcohol Use (Ref=No) | 10781 | 1.85 (1.70-2.02) | <0.001 | 0.97 (0.87 1.08) | 0.584 |
| Age at First Use of Alcohol  10-19  20-29  30+  Never | 7486 | 1.22 (0.80-1.85)  1.54 (1.02-2.33)  1.36 (0.89 2.10)  Ref | 0.568  0.001  0.317 | 0.97(0.59-1.61)  1.01 (0.61-1.65)  0.86 (0.52-1.43)  Ref | 0.860  0.477  0.206 |
| Total Number of Children  Less or equal 2  Less or equal 4  Equal or more than 5  None | 10794 | 15.75 (12.87-19.27)  21.36 (17.42-26.20)  18.79 14.67-24.06)  Ref | <0.000  <0.000  <0.000 | 9.28 (7.25-11.87)  13.6 (10.51-17.60)  12.34(9.149 16.65)  Ref | **<0.000**  **<0.000**  **<0.000** |
| Age of Husband  15-24  25-34  35+ | 7960 | 0.69 (0.55-0.87)  1.09 (1.01-1.21)  Ref | 0.003  0.001 | 0.83 (0.60-1.15)  0.97 (0.82-1.13)  Ref | 0.278  0.512 |
| Area of Residence  Urban  Rural | 11737 | 0.64 (0.60- 0.70)  Ref | <0.000 | 0.69 (0.62-0.78)  Ref | **<0.000** |
| Region of Origin  Khangai  Central  Eastern  Ulaanbaatar  Western | 11737 | 1.35 (1.21-1.52)  1.08 (0.95-1.22)  1.29 (1.13-1.47)  0.81 (0.72-0.90)  Ref | <0.000  0.846  0.003  <0.000 | 1.35 (1.19-1.53)  1.08 (0.94-1.25)  1.31 (1.13-1.51)  1.05 (0.91-1.21)  Ref | **0.000**  0.225  **0.008**  **0.051** |
| Ethnicity  Khalkh  Kazakh  Other | 11737 | 1.08 (1.01-1.214)  0.95 (0.80-1.12)  Ref | 0.028  0.184 | 1.15 (1.02-1.30)  1.44 (1.01-2.04)  Ref | **0.052**  **0.008** |
| Religion  Buddhist  Islam  Other  No Religion | 11737 | 0.95 (0.88-1.06)  0.81 (0.70-0.93)  0.78 (0.63-0.96)  Ref | 0.086  0.139  0.115 | 0.95 (0.87 1.03)  0.69 (0.49- 0.98)  0.86 (0.69-1.06)  Ref | 0.129  **0.010**  0.928 |
| Wealth Index Score  Richest  Fourth  Middle  Second  Poorest | 10794 | 0.67 (0.58-0.76)  0.68 (0.60-0.77)  0.65 (0.58-0.73)  0.76 (0.68-0.85)  Ref | **0.038**  **0.051**  **0.001**  **0.052** | 0.97 (0.85-1.10)  0.85 (0.74-0.97)  0.90 (0.78-1.03)  0.99 (0.83-1.17)  Ref | 0.438  **0.018**  0.302  0.349 |
| OR: Odds Ratio  AOR: Adjusted Odds Ratio (stepwise adjusted for individual and community level factor)  95% CI: 95% Confidence Interval | | | | | |
